# Assessing the sensitivity and specificity of First Response HIV-1-2 test kit with whole blood and serum samples: a cross-sectional study

**DOI:** 10.1186/s12981-016-0092-0

**Published:** 2016-02-16

**Authors:** Raymond Boadu, George Darko, Priscilla Nortey, Patricia Akweongo, Bismark Sarfo

**Affiliations:** Tamale Central Hospital, Ministry of Health, Tamale, Ghana; Department of Epidemiology and Disease Control, School of Public Health, College of Health Sciences, University of Ghana, Accra, Ghana

**Keywords:** HIV Rapid Diagnostic test, First Response HIV-1-2 test kit, HIV testing, Sensitivity, Specificity, Predictive values, Blood-specimen

## Abstract

**Background:**

Human immunodeficiency virus (HIV) Rapid diagnostic Test (RDT) kits are the preferred assays for HIV testing in many countries. Prevention of Mother-to-Child Transmission, Know Your Status Campaigns, Blood-Safety, Voluntary Counseling and Testing are major strategies adapted to control transmission of the virus and the pivot of these interventions is either screening or diagnosing individuals through testing. There are reports of inconsistent sensitivity and specificity with whole blood and serum samples collected from the same individual. Little is known about the diagnostic characteristics of First Response HIV-1-2 RDT kit, used as a single test kit in national HIV prevention and control programmes. The debate has always centered on choosing between whole blood and serum in a case where a single test kit that runs on only blood specimen will be used for testing. The variations in specificities and sensitivities with whole blood and serum samples imply that some individuals who might be true positives will be missed and elude care. This study determined the best blood-based specimen type (whole blood or serum) that improves performance of First Response HIV RDT kit in detecting HIV-specific antibodies.

**Methods:**

A hospital-based cross-sectional study was conducted on 280 HIV infected and non-infected patients from May 2015 to June 2015. Blood samples from each participant were separated into whole blood and serum, and tested on First Response HIV-1-2 kits (Premier Medical Corporation Ltd., Kachigam, India) using Electro-chemi-luminescence assay (ECLIA) as reference assay.

**Results:**

First Response HIV-1-2 RDT kit showed 100 % sensitivity and 100 % specificity with whole blood specimen and 100 % sensitivity and 82.86 % specificity with serum specimen for the detection of HIV-1. The positive and negative predictive values were 100, 100 and 85.35, 82.86 % for whole blood and serum respectively.

**Conclusion:**

Whole blood specimen(s) from an individual have higher specificity, positive and negative predictive values than serum. Whole blood is the primary specimen to use on First Response HIV-1-2 RDT kit when screening peripheral blood for HIV-1-specific antibodies.

## Background

Diagnosing Human Immuno-deficiency Virus (HIV) infection at the early stages is instrumental in HIV/AIDS disease surveillance. Technologies that allow the detection of very low levels of HIV nucleic acids (RNA or DNA), viral p24 antigens, and HIV-specific antibodies have been developed [1]. In spite of these successes, laboratory assays in general are still imperfect, and some recent infections remain undetected [2]. Viral load test, the gold standard, can identify 96 % of recent infections. The fourth generation Enzyme Immuno Assays (EIAs) identify 93 % and the third generation EIAs [Rapid Diagnostic Test (RDT) kits] (also identify) 63 % of all recent infections [3]. Although RDT kits have the lowest detection ability, they remain the preferred assay for HIV testing in many countries. This is because they require little expertise to use, run on little sample volumes, cheaper in cost and produce test results in 15 min [4]. The performance of HIV RDT kits differ with brand(s) [5], therefore, are used in combination to improve diagnosis [6]. Ghana has adopted the serial testing algorithm where First Response HIV-1-2 kit (Premier Medical Corporation Ltd., Kachigam, India) is used to screen samples and Oraquick Advance HIV-1-2 kit (OraSure Technologies, Bethlehem, PA, USA) is primarily used to confirm infection. Nonetheless, First Response HIV-1-2 kit is often used as a single test kit in national HIV prevention and control programmes. A study by Laperche et al. [7], linked the practice of single RDT kit use (for screening test samples) to high HIV infection rates. In that study, single RDT kit use detected 85 % of infections that were detectable by standard Enzyme Linked Immuno-Sorbent Assay (ELISA) [7]. In other words, 15 % HIV infected individuals were not detected when a single RDT kit was used to screen the population. The results were similar when the test specimen was either whole blood or serum.

Blood specimen (whole blood, plasma or serum) is the sample to apply on First Response HIV-1-2 RDT kit. Field reports indicate that blood specimen contains unequal concentrations of detectable HIV-specific antibodies in whole blood and serum. Sensitivity and specificity of HIV rapid test kits differ with whole blood (95 %) and serum specimens (98 %) [6, 8]. Better specificity (99.9 %) is achieved with whole blood than using serum specimen (99 %) [9]. One may argue that it is better to use highly sensitive samples (serum) for testing. Conversely, a field study found serum samples to produce more false negative results compared to whole blood samples [10]. This loss of sensitivity is important especially in resource limited settings where RDTs are widely used. A study in Cape Town, South Africa, recorded 1100 HIV positive cases that were initially diagnosed negative due to poor RDT sensitivity [5]. Other studies conducted in sub-Saharan Africa reported that 3 % of people undergoing HIV testing at out-patient departments are at risk of receiving false negative results and recommended a follow-up post-transfusion HIV testing for blood recipients to monitor sero-conversion [11, 12].

The concern of the authors suggests that the test specimen and/or kit used to screen blood prior to donation might have missed possible positive cases.

Owusu-ofori et al. [13], proposed that Ghana complement serologic rapid test with Nucleic Acid Test (NAT) in pre-donation screening of blood donors. Nevertheless, NAT comes with a lot of technical and economic constraints. This leaves the country in equipoise since studies on the affordable HIV RDT kits are reporting varying sensitivity and specificity with test specimen types. The evidence from these studies suggests a need to assess HIV RDT kits used in specific settings to determine the blood-based specimen type that improves performance. The dilemma now is deciding between whole blood and serum in cases where single test kits are used for testing.

In Ghana, depending on the setting where the test is performed, either whole blood or serum sample is used on the First Response HIV-1-2 kit. What is not known is whether the test kit produces consistent results with these specimens and there have not been any independent post-market assessment studies to ascertain the comparability of the kit using the different blood-based specimens. This study assessed the First Response HIV-1-2 kit using whole blood and serum samples to determine its performance characteristics in these specimens.

## Results

### Clinical samples processing

In this study, we did not detect HIV-2 among the samples tested. 280 out of the 295 (94.9 %) samples collected were accurately classified. All 140 (50 %) samples collected from already diagnosed patients under treatment tested positive for HIV-1. 2 OPD and three prospective blood donor samples were positive for HIV-1 but they were excluded from the analysis. The OPD patients were referred to the ART centre for counseling to commence treatment. The decision on the blood donors was referred to the Regional Blood Service.

140 (50 %) samples were negative for HIV after testing. 100 were collected from prospective blood donors and 40 from OPD patients. 35 (12.5 %) samples produced discordant results.

25 out of the 35 discordant samples (71.43 %) were classified as negative according to the criterion described later. These samples produced non-reactive results for both whole blood and serum on the test kit and non-reactive serum result on the analyzer (ECLIA). 10 samples out of the 35 discordant samples (28.57 %) produced discordant results after repeated tests and could not be classified. They were excluded from the analysis.

### Proportion of HIV-1 infected and non-infected people testing positive or negative with serum on First Response HIV-1-2 RDT kit and ECLIA

First Response HIV-1-2 RDT kit accurately identified all 140 HIV-1 infected samples as positive when serum was used as the test specimen. The test kit achieved comparable sensitivity (100 %) with ECLIA. The kit’s positive predictive value using serum as test specimen was 100 % (Table 1). 116 of the 140 HIV-1 non-infected samples tested negative using serum as test specimen on First Response HIV-1-2 kit. 24 samples tested positive (false positive) and did not agree with the negative results obtained from ECLIA. The specificity of the test kit was 82.86 % and was significantly lower compared to ECLIA (100 %) as depicted in Table 2.

**Table 1:** Sensitivity, positive predictive value (PPV) of assays with serum specimen

Test (assay)	No. of HIV-1 positive samples	Positive serum results	False negative result	Sensitivity (%)	PPV (%)
First response HIV-1-2 kit	140	140	0	100	100
ECLIA (reference)	140	140	0	100	100

**Table 2:** Specificity, negative predictive value (NPV) of assays with serum specimen

Test (assay)	No. of HIV-1 negative samples	Negative serum results	False positive result	Specificity (%)	NPV (%)	*P* value
First response HIV-1-2 kit	140	116	24	82.86	82.86	<0.001
ECLIA (reference)	140	140	0	100	100	

### Proportion of HIV-1 infected and non-infected people testing positive or negative with whole blood on First Response HIV-1-2 kit and ECLIA

Using whole blood as test specimen, First Response HIV-1-2 RDT kit accurately detected all 140 HIV-1 positive samples collected from 140 HIV-1 infected people. The kit was 100 % sensitive and 100 % positive prediction in detecting HIV-1 that agreed with ECLIA (Table 3). This was not contrary to what was indicated on the test kit (99.8 %).

**Table 3:** Sensitivity, positive predictive value (PPV) of assays with whole blood specimen

Test (assay)	No. of HIV-1 positive samples	Positive whole blood results	False negative result	Sensitivity (%)	PPV (%)
First response HIV-1-2 kit	140	140	0	100	100
ECLIA (reference)	140	140	0	100	100

All the 140 HIV non-infected samples that tested negative on First Response HIV-1-2 RDT kit using whole blood as test specimen agreed with the ECLIA results. The test kit was as good as ECLIA showing 100 % for specificity and negative predictive value (Table 4).

**Table 4:** Specificity, negative predictive value (NPV) of assays with whole blood specimen

Test (assay)	No. of HIV-1 positive samples	Negative whole blood results	False positive result	Specificity (%)	NPV (%)
First response HIV-1-2 kit	140	140	0	100	100
ECLIA (reference)	140	140	0	100	100

### Proportion of HIV infected and non-infected people testing positive or negative with serum or whole blood on First Response HIV-1-2 RDT kit compared to ECLIA

Overall, First Response HIV-1-2 RDT kit detected HIV-1 antibodies in 164 of the 280 samples collected from both HIV-1 positive and negative patients as positive with serum specimen (Table 5). 116 of the 280 samples were identified as negative with serum specimen (Table 5). 140 HIV-1 positive samples were detected as positive and 140 samples were identified as negative using whole blood as test specimen on First Response HIV-1-2 RDT kit (Table 5). ECLIA detected 140 of the 280 samples as positive and 140 as negative. 24 false positives results were produced with serum specimen. ECLIA demonstrated 100 % sensitivity and specificity, and 100 % positive and negative predictions (Table 5).

**Table 5:** Sensitivity, specificity, positive and negative predictive values of First response HIV-1-2 kit compared to ECLIA

Test-type and specimen	Test results		False positive	False negative	No. of samples	Sensitivity (%)	Specificity (%)	PPV (%)	NPV (%)	P value
	Positive	Negative								
Whole blood	140	140	0	0	280	100	100	100	100	
Serum	164	116	24	0	280	100	82.86	85.37	82.86	<0.001
ECLIA (reference)	140	140	0	0	280	100	100	100	100	

First Response HIV-1-2 RDT kit showed the same sensitivity (100 %) as ECLIA with both whole blood and serum specimens but significantly lower specificity (82.86 %) with serum specimen (p < 0.001). The predictive values were also significantly lower with serum specimen compared to whole blood and ECLIA for positivity (85.37 %) and negativity (82.86 %) (p < 0.001). All the positive results showed strong reactive bands and there were no weak reactive results (Table 5).

## Discussion

Collectively, First Response HIV-1-2 RDT kit showed 100 % sensitivity for the detection of HIV-1 with both serum and whole blood specimens, 100 % specificity with whole blood and these results completely agreed with those of ECLIA. Yet, the high number of False Positives (25) observed with serum specimen on the test kit was contrary to ECLIA and resulted in a lower Positive and Negative Predictive Values (85.37 %) (82.86 %) respectively. These results were consistent with Anzala et al. [14] who assessed Rapid Diagnostic Test (RDT) kits used for counseling and testing in Kenya and Uganda.

Positive Predictive Value (PPV) is the proportion of patients with a positive test who actually have the disease. Negative Predictive Value (NPV) is the proportion of patients with a negative test who do not have the disease. One way to avoid confusing this with sensitivity and specificity is to imagine that you are a patient and you have just received the results of your screening test. If the test was positive, then what is the probability that ‘you’ the patient really have the disease (Positive Prediction), that is how worried should you be? Conversely, if it is good news, and the screening test was negative, how reassured should you be? Thus the probability that you are disease free? (Negative Prediction).

Generally, the performance of HIV rapid diagnostic testing in a population is influenced by Positive and Negative Predictive values of the RDT kits used. Thus sensitivity and specificity results obtained from test kit evaluation studies (quoted in manufacturer’s instruction manuals) prior to licensing and marketing of the kit will not necessarily be achieved in practice. Predictive values vary among populations such that PPV and NPV of HIV RDT kits are lower in low HIV prevalent areas like Ghana (1.3 %) [15]. In this study a PPV of 85.37 % was recorded with serum specimen explaining the higher False Positives and the lower negative prediction of the test kit compared to ECLIA. This suggests that ~15 % of people who test with serum specimen on First Response HIV-1-2 RDT kit in such study settings with similar characteristics as Ghana will receive False Positive results. Among the positive samples tested, both whole blood and serum showed similar PPVs (100 %) as ECLIA. This suggests however, that First Response HIV-1-2 RDT kit is well designed to detect HIV-1 antibodies in blood specimen if they so contain any. Never the less, the lower specificity (82.86 %) expressed by the RDT kit indicates the occurrence of high cross-reactivity when serum is used as the test specimen. Serum specimens are known to contain various antibodies that may bind to reaction sites (epitopes) meant for target antibodies [16]. Therefore, First Response HIV-1-2 RDT kits may present the challenge of high antibody cross-reactivity with serum specimen than whole blood. Hence, serum specimen may not accurately predict the presence or absence of HIV-1-specific antibodies in the blood. The increased PPV and NPV with whole blood in the current study may imply that the lower performance of the test kit observed under serum could be improved with whole blood specimens. Accordingly, a second test kit of higher specificity may not be needed to confirm serum results on First Response HIV-1-2 RDT kit as recommended by Parekh et al. [15]. In the case of Ghana, this will save cost, and the more expensive and scarce OraQuick Advanced HIV-1-2 RDT kits may not be fast needed to confirm initially screened serum results. Alternatively, samples may be screened first with whole blood instead of serum on First Response HIV-1-2 RDT kit and reactive ones confirmed on OraQuick Advance HIV-1-2 RDT kit to minimize wastage of the later.

Although the comparable sensitivity and PPV results (100 %) achieved with First Response HIV-1-2 RDT kit in the current study using whole blood differed in absolute percentages from Kroidl et al. [10] (99.5 %) yet were consistent with those reported by the manufacturer (100 %). Moreover, this study tested whole blood samples collected from low HIV prevalent populations whereas Kroidl et al., 2012, sampled the blood from high prevalent populations. This gives a hint to better use whole blood on First Response HIV-1-2 RDT kit in both low and high HIV prevalent populations. Even though the higher Positive Predictive Value and zero False Positive results achieved with whole blood specimen in this study were contrary to what was reported by Bi et al. [17]; the two studies differ in certain respect. Whereas First Response HIV-1-2 RDT kit was used in the current study, OraQuick Advance HIV-1-2 RDT kit was assessed in theirs. Notwithstanding, it has been documented by Moal et al. [6] that HIV RDT kits from different manufacturers vary in sensitivity and specificity. Providing grounds to infer that the technologies used to design these kits may also differ hence would not yield parallel results with similar test specimen.

### Limitations and strengths of the study

The present study is limited in various ways, and that the results should be interpreted with caution. Commercial ELISA that has been fully described in the study setting was not used as the reference test. There were no documents in the current setting that compared the HIV diagnostic performance of ECLIA to commercial ELISA. Nonetheless, the diagnostic successes of ECLIA over commercial ELISA [17] cannot be ignored.

This study is one of the few that assessed HIV RDT kits against ECLIA [17, 18] and the only one that had compared First Response HIV-1-2 RDT kit to ECLIA. Again, the False Positive results observed with serum specimen were not confirmed with PCR because of limited logistics and technical constraints.

Nevertheless, ECLIA is highly sensitive and specific and had performed well against PCR in diagnosing HIV infection in multiethnic regions [17]. Moreover, all the False Positive serum samples were negative with whole blood and this was consistent with ECLIA which also uses serum as test specimen. These results show the success of the exclusion criteria since many discrepant results were minimized.

## Conclusion

Using serum as test specimen on First Response HIV-1-2 RDT kit increases the tendency of producing false positive results. Whole blood specimens from individuals have higher specificity, and positive and negative predictive values than serum. Whole blood is the primary specimen to use on First Response HIV-1-2 RDT kit when screening peripheral blood for HIV-1-specific antibodies.

### Recommendation

Whole blood specimen is excellent to use on First response HIV-1-2 RDT kit to screen populations in Ghana to detect HIV-1-specific antibodies. Whole blood should be preferred to serum specimen in blood banks to screen prospective donors. Settings that use serum as test specimen should adopt whole blood but must confirm all reactive results with standard confirmatory tests. Future studies should assess the diagnostic characteristics of the test kit with whole blood and serum samples of HIV-2 positive people.

## Methods

### Population

The study was conducted in three different hospitals (Tamale Teaching Hospital, Tamale Central Hospital and Savelugu Municipal Hospital) in the northern region of Ghana.

### Ethnicity

The main ethnic group in Tamale metropolis and Savelugu municipal is Dagomba. However other ethnic groups such as Akan, Frafras, Mamprusi, Moshe, Ewe, can be found in both the metropolis and the municipality [19].

### Socio-economic status

The major occupation(s) in these areas (Tamale Metropolis and Savelugu municipality) are farming, craftsmanship and petty trading among women and men. A proportion of the population engages in public services, however, unemployment rate among the youth of the metropolis is high, reflecting the high poverty level in the metropolis.

### Participants

Participants were recruited from the Out-Patient Department (OPD), laboratory and Anti-Retro Viral Therapy (ART) centres of these hospitals. They involved: adult HIV patients (≥18 years) who had been on antiretroviral therapy for not more than 10 years; adult OPD patients (≥18 years) with positive or negative HIV results after laboratory testing; patients with normal haematological and biochemical test results after laboratory analysis; and prospective blood donors who tested either positive or negative for HIV, Hepatitis B, Hepatitis C and syphilis. In order to minimize false positive and negative results, all adult HIV patients (≥18 years) who had received antiretroviral therapy for more than 10 years were excluded [11]. Also, all pregnant women who visited the facilities were not considered as participants for this study [14]. Additionally, children less than 18 months born to HIV positive mothers were not recruited [20].

### Sample size calculation

The sample size (N) was calculated using the sensitivity (100 %) and specificity (99.5 %) values quoted in the test kit manufacturer’s instruction package insert and the national HIV prevalence (1.3 %). These were imputed into the formula, $({\text{PPV}} = \frac{{{\text{sensitivity }} \times {\text{prevalence}}}}{{{\text{sensitivity }} \times {\text{prevalence}}}} + (1 - {\text{prevalence}})$ and ${\text{NPV}} = \frac{{{\text{specificity }} \times (1 - {\text{prevalence}})}}{{(1 - {\text{sensitivity}}) \times {\text{prevalence}}}} + {\text{specificity}} \times (1 - {\text{prevalence}})$ to estimate the positive and negative predictive values of the test kit. The positive predictive value was 97 % and the negative predictive value was 100 %. This means there is a 3 % probable positive case that will not be detected by the kit. The predictive values were computed in a sample size calculator to determine the sample size by the formula N = 2 {10.5 × [PPV × (100−NPV) + NPV × (100−PPV)/(PPV−NPV) ^2]} at a power of 80 % and a significance level of 0.05. The sample size was 280 participants after correcting for non-respondents. 15 (5.08 %) participants had incomplete data and were categorized as non-respondents.

### Study design

A hospital-based cross-sectional study was conducted on HIV infected and non-infected patients from May 2015 to June 2015. Whole blood and serum samples collected from these participants were tested on First Response HIV-1-2 RDT kit, and ECLIA technology was used as the gold standard assay. The test kit’s sensitivity, specificity, positive and negative predictive values achieved with serum and whole blood specimens were determined and compared.

### Testing procedures

#### Clinical samples collection, processing and storage

The specimens used in this study were fresh samples from HIV infected patients, OPD patients and prospective blood donors. Fresh sets of samples (EDTA-anti-coagulated whole blood, and serum) were collected from 295 patients (This includes participants with incomplete data).

Venous blood collected from each participant was divided into EDTA-anticoagulant and serum separator tubes labelled with the patient’s identification. The anticoagulant prevented the blood from clotting making it possible to obtain whole blood.

The serum separator tubes were centrifuged at 3000 rpm for 15 min to obtain the serum samples. The whole blood samples were stored at refrigeration temperature at 4 °C and the serum samples at −20 °C until used. Testing was done not more than 72 h after sample collection.

#### HIV antigen (p24)/antibody test

HIV Antigen (p24)/antibody tests were done by automation on Cobas E 411 analyzer using serum samples. Because the sample of choice for this analyzer was serum, whole blood samples could not be analyzed. All 295 serum samples were analyzed to determine their HIV sero-status.

#### Cobas E 411

Cobas E 411 (Roche Diagnostics GmbH, Mannheim, Germany) is a fully automated immunoassay analyzer which uses electro-chem-iluminescence immunoassay (ECLIA) technology to detect HIV-1 and HIV-2 in human serum. The analyzer had sample loading chambers, a touch-screen monitor and a printer. This study used Elecsys HIV combi PT (Roche Diagnostics GmbH, Mannheim, Germany) as reagents. This reagent is a fourth generation qualitative immunoassay for the determination of HIV-1 p24 antigens and antibodies to HIV-1, including group O, and HIV-2 in serum. Test results were produced within 30 min after loading samples.

Samples with cutoff index (COI) <0.9 were considered non-reactive (negative) and those with COI ≥1.0 considered reactive (positive). There were no observed COI readings in the range ≥0.9 to <1.0 (borderline) among the samples, HIV RNA test was therefore not needed.

#### HIV antibody test

All HIV antibody tests were performed by applying whole blood or serum on First Response HIV-1-2 test kits.

#### First Response HIV-1-2 test kit

First Response HIV-1-2 (Premier Medical Corporation Ltd., Kachigam, India) kit is a rapid immuno-chromatographic qualitative test for the detection of antibodies to HIV-1 and HIV-2 in whole blood, plasma or serum. The kit was packaged with a sample pipette and a sample buffer.

The kit had two test-band regions. Region “1” was pre-coated with recombinant HIV-1 antigens (gp 41, including group O and p24) and region “2” was pre-coated with recombinant HIV-2 antigen (gp36). These recombinant antigens were conjugated with colloidal gold particles. There was a region labelled “C”, the inbuilt control line which indicated successful antigen–antibody reaction.

The results were interpreted according to the manufacturer’s instruction document. Red lines at both regions “1” and “C” meant HIV-1 infection and at regions “2” and “C” was indicative of HIV-2 infection.

Legend negative (single red band at control line). Positive (two red bands, one at 1 and the other at the control).

#### Classifying samples as HIV infected or non-infected

We used ECLIA technology as the gold standard test in this study. The whole blood and serum samples were first tested on First Response HIV-1-2 test kits simultaneously. Both reactive and non-reactive samples were retested on Cobas E 411 analyzer. This enabled us to classify the samples as HIV infected and HIV non-infected. Samples with reactive results for both whole blood and serum on the test kit and reactive serum result on the analyzer were classified positive.

Samples with non-reactive results for both whole blood and serum on the test kit and non-reactive serum result on the analyzer were classified negative. For samples that produced results on the test kit which did not agree with the analyzer (discordant results), a decision was made after consultations with HIV diagnostic experts. A repeated test was done with the test kit. The two results (initial and repeated) were compared and the blood-based specimen type (whole blood or serum) result that agreed with the analyzer was chosen as the final result for classification.

All patient test results and information were treated confidential and the study was approved by the Ethical Review Board of Ghana Health Service (approval number GHS-ERC: 06/02/15).

### Data analysis

Data analyses were performed using the statistical software, StataSE (version 13.1, StataCorp, Texas, USA). Variables were summarized as frequencies and percentages with significant level at 0.05. Sensitivity, specificity, positive and negative predictive values were determined using cross-tables and standard formulas {(PPV = sensitivity × prevalence/sensitivity × prevalence + (1−prevalence)} and {(NPV = specificity × 1−prevalence)/(1−sensitivity) × prevalence + specificity × (1−prevalence)}.

## Abbreviations

AIDS: Acquired Immuno Deficiency Syndrome
ART: antiretroviral therapy
DNA: deoxyribonucleic Acid
ECLIA: Electro-Chemi-Luminesence Assay
EIA: enzyme Immuno Assay
ELISA: enzyme Linked Immuno Sorbent Assay
FP: false Positive
HIV: human Immuno Deficiency Virus
LDF: laboratory Data Form
NACP: national AIDS Control Programme
NAT: nucleic Acid Test
NGO: non-Governmental Agency
OPD: out-Patient Department
PPV: positive Predictive Value
NPV: negative Predictive Value
RDT: rapid Diagnostic Test
RNA: ribonucleic Acid

## References

[1] Buttò S, Suligoi B, Fanales-belasio E (2010). Laboratory diagnostics for HIV infection. Ann Ist Super Sanita.

[2] Cohen MS, Gay CL, Busch MP, Hecht FM (2010). The detection of acute HIV infection. J Infect Dis.

[3] Branson BM (2006). To screen or not to screen: is that really the question?. Ann Intern Med.

[4] Arai H, Petchclai B, Khupulsup K, Kurimura T, Takeda K (1999). Evaluation of a Rapid Immunochromatographic Test for Detection of Antibodies to Human Immunodeficiency Virus. J Clin Microbiol.

[5] Wolpaw BJ, Mathews C, Chopra M, Hardie D, de Azevedo V, Jennings K, Lurie MN (2010). The failure of routine rapid HIV testing: a case study of improving low sensitivity in the field. BMC Health Services Res.

[6] Moal L, Saberan-Roncato M, Plainchamp D, Hocqueloux L, Prazuck T, Camps P (2014). Finger-Stick Whole Blood HIV-1/-2 Home-Use Tests Are More Sensitive than Oral Fluid-Based In-Home HIV Tests. PLoS ONE.

[7] Laperche S, Leballais L, Ly TD, Plantier JC (2012). Failures in the detection of HIV p24 antigen with the Determine HIV-1/2 Ag/Ab Combo rapid test. J Infect Dis.

[8] Pavie J, Rachline A, Loze B, Niedbalski L, Delaugerre C, Laforgerie E, Plantier JC, Rozenbaum W, Chevret S, Molina JM, Simon F (2010). Sensitivity of five rapid HIV tests on oral fluid or finger-stick whole blood: a real-time comparison in a healthcare setting. PLoS ONE.

[9] Lyamuya EF, Aboud S, Urassa WK, Sufi J, Mbwana J, Ndugulile F, Massambu C (2009). Evaluation of simple rapid HIV assays and development of national rapid HIV test algorithms in Dar es Salaam, Tanzania. BMC Infect Dis.

[10] Kroidl I, Clowes P, Mwalongo W, Maganga L, Maboko L, Kroidl AL, Geldmacher C, Machibya H, Hoelscher M, Saathoff E (2012). Low specificity of determine HIV1/2 RDT using whole blood in south west Tanzania. PLoS ONE.

[11] Bassett IV, Chetty S, Giddy J, Reddy S, Bishop K, Lu Z, Losina E, Freedberg KA, Walensky RP (2011). Screening for acute HIV infection in South Africa: finding acute and chronic disease. HIV Med.

[12] Kubio C, Tierney G, Quaye T, Nabilisi JW, Ziemah C, Zagbeeb SM, Shaw S, Murphy WG (2012). Blood transfusion practice in a rural hospital in Northern Ghana, Damongo, West Gonja District. Transfusion.

[13] Owusu-Ofori S, Temple J, Sarkodie F, Anokwa M, Candotti D, Allain JP (2005). Predonation screening of blood donors with rapid tests: implementation and efficacy of a novel approach to blood safety in resource-poor settings. Transfusion.

[14] Anzala O, Sanders EJ, Kamali A, Katende M, Mutua GN, Ruzagira E, Stevens G, Simek M, Price M (2008). Sensitivity and specificity of HIV Rapid Tests used for Research and Voluntary Counselling and Testing, Determine and Capillus. East Af Med J.

[15] Parekh BS, Kalou MB, Alemnji G, Ou C, Nkengasong JN (2010). Scaling Up HIV Rapid Testing in Developing Countries Comprehensive Approach for Implementing Quality Assurance. Am J Clin Pathol.

[16] Abokyi LV, Zandoh C, Mahama E, Sulemana A, Adda R, Baiden F (2014). Willingness to undergo HIV Testing in the Kintampo District of Ghana. Ghana Med J.

[17] Bi X, Ning H, Wang T, Li D, Liu Y, Yang T, Yu J, Tao C (2012). Comparative Performance of Electro chemiluminescence Immunoassay and EIA for HIV Screening in a Multiethnic Region of China. PLoS ONE.

[18] Black V, von Mollendorf CE, Moyes JA, Scott LE, Puren A, Stevens WS (2009). Poor sensitivity of field rapid HIV testing: implications for mother-to-child transmission programme. BJOG: An Int J Obstet Gynaecol.

[19] Ghana Statistical Service. Population and Housing Census Summary Report of Final Results. 2012; pp.90–117.

[20] Claassen M, van Zyl GU, Korsman SN, Smit L, Cotton MF, Preiser W (2006). Pitfalls with rapid HIV antibody testing in HIV-infected children in the Western Cape, South Africa. J Clin Virol.

